# Nasopharyngeal pneumococcal carriage and serotype landscape in children, adolescents and young adults in Türkiye

**DOI:** 10.1007/s00431-026-06744-6

**Published:** 2026-01-22

**Authors:** Mahmut Can Kizil, Omer Kilic, Yalcin Kara, Mucahit Kaya, Adem Karbuz, Merve Iseri Nepesov, Ergin Ciftci, Halil Ozdemir, Fatma Nur Oz, Zafer Kurugol, Solmaz Celebi, Benhur Sirvan Cetin, Dilek Yilmaz, Meltem Dinleyici, Didem Kizmaz Isancli, Onder Kilicaslan, Rabia G. Sezer Yamanel, Belkıs Hatice Inceli, Dondu Nilay Penezoglu, Burce Dortkardesler, Fatma Dilsad Aksoy, Sedanur Tekin Can, Nesli Agrali Eroz, Ener Cagri Dinleyici

**Affiliations:** 1 Department of Pediatric Infectious Disease, Faculty of Medicine, Eskisehir Osmangazi University, Eskisehir, Türkiye; 2 AD Genetics, Ankara, Türkiye; 3 Department of Pediatric Infectious Disease, Dr. Cemil, Tascioglu City Hospital, Istanbul, Türkiye; 4 Department of Pediatric Infectious Disease, Zeynep Kamil Maternity and Children Hospital, Istanbul, Türkiye; 5 Department of Pediatric Infectious Disease, Faculty of Medicine, Ankara University, Ankara, Türkiye; 6 Department of Pediatric Infectious Disease, Ankara Etlik State Hospital, Ankara, Türkiye; 7 Department of Pediatric Infectious Disease, Faculty of Medicine, Ege University, Izmir, Türkiye; 8 Department of Pediatric Infectious Disease, Faculty of Medicine, Uludag University, Bursa, Türkiye; 9 Department of Pediatric Infectious Disease, Faculty of Medicine, Erciyes University, Kayseri, Türkiye; 10 Department of Pediatric Infectious Disease, Faculty of Medicine, Izmir Katip Celebi University, Izmir, Türkiye; 11 Department Social Pediatrics, Faculty of Medicine, Eskisehir Osmangazi University, Eskisehir, Türkiye; 12 Department of Pediatrics, Faculty of Medicine, Eskisehir Osmangazi University, Eskisehir, TR-26040 Türkiye

**Keywords:** Pneumococci, Carriage, Conjugated pneumococcal vaccine

## Abstract

After the widespread use of pneumococcal conjugated vaccines (PCVs), pneumococcal carriage, especially due to some vaccine serotypes, has been shown to decrease, but carriage with non-vaccine serotypes and some persistent vaccine types of lineages has been demonstrated to continue. Evaluation of pneumococcal carriage helps to understand disease epidemiology. In this multicenter study, we aimed to determine pneumococcal carriage and serotype distribution in children, adolescents, and young adults aged 0–24 years in Türkiye after the pandemic era. This multicenter study was conducted between April and August 2022 in 1585 healthy children, adolescents, and young adults (aged between 0 and 24 years) in nine centers in Türkiye. Demographics, schooling/day‑care, smoking exposure, recent upper respiratory tract infection (URTI), antibiotic use (1 and 3 months), COVID‑19 infection/vaccination, and pneumococcal vaccination history were recorded. Nasopharyngeal swab samples were taken from all participants. *Streptococcus pneumoniae* was detected by real‑time polymerase chain reaction (PCR); positives were serotyped by singleplex real‑time PCR assays targeting 33 serotypes/serogroups. Among 1 585 participants (797 female; age distribution 0–5 years 22.0%, 6–10 years 29.3%, 11–15 years 16.8%, 16–18 years 12.9%, 19–24 years 19.0%), overall pneumococcal carriage prevalence was 19.6% (311/1 585). Age‑specific prevalences were 20.7% (0–5 years), 21.8% (6–10 years; peak), 19.1% (11–15 years), 15.6% (16–18 years), and 18.2% (19–24 years). Two‑thirds (66.2%) had received ≥ 1 PCV dose (coverage ≥ 82% through 15 years, declining to 43.9% at 16–18 years and 13.3% at 19–24 years). Vaccination was associated with significantly lower carriage only in children ≤ 10 years: 0–5 years 17.8% vs 43.6% (OR 0.28, 95% CI 0.13–0.60, *p* < 0.001); 6–10 years 19.7% vs 32.4% (OR 0.51, 0.28–0.93, *p* = 0.021). No significant differences were seen in older strata or overall (18.8% vs 21.3%, OR 0.85, 0.65–1.12). Of 311 isolates, 225 (72.4%) were typed (27 serotypes) and 86 (27.6%) were not defined. Dominant serotypes were 19F, 6A/B, 3, 23F, and 15B/C; PCV13 serotypes comprised 77.3% of typed isolates. Theoretical vaccine coverage among 225 typed isolates increased from 61–64% (PCV7/10) to 77.3% (PCV13), 78.2% (PCV15), 88.4–90.2% (PCV20/24), plateauing at 93.3–93.8% for PCV31/25. Theoretical vaccine coverage in children aged below 5 years of age was 66.7% for PCV13, 70.0% for PCV15, and 88.3% for PCV20. The frequency of PCV13 serotypes in children vaccinated with PCV13 was significantly lower than in unvaccinated children in children below 5 years of age.

*Conclusion*: Post‑pandemic pneumococcal carriage in Türkiye remains 19.6% across childhood. Direct protection against nasopharyngeal carriage was evident in children ≤ 10 y. Higher‑valency PCVs and enhanced genomic serotype surveillance are needed to address residual carriage and guide future immunization strategies.

**Table:** 

**What is Known:** • *Pneumococcal conjugate vaccines (PCVs) have substantially reduced invasive pneumococcal disease, but nasopharyngeal colonization persists due to serotype replacement.* • *After the COVID-19 pandemic, major shifts in respiratory pathogen epidemiology occurred, yet contemporary post-pandemic data on pneumococcal carriage and serotype distribution remain scarce.*
**What is New:** • *This is the first multicenter post-COVID pneumococcal carriage study in Türkiye covering the full 0–24-year age spectrum, showing that carriage remains stable at ~20%.* • *Direct vaccine protection against carriage is confined to children ≤10 years, with no measurable impact in adolescents or young adults. Some vaccine serotypes and non-vaccine serotypes still dominate carriage, and higher-valency PCVs would markedly improve theoretical coverage.*

**What is Known:**

• *Pneumococcal conjugate vaccines (PCVs) have substantially reduced invasive pneumococcal disease, but nasopharyngeal colonization persists due to serotype replacement.*

• *After the COVID-19 pandemic, major shifts in respiratory pathogen epidemiology occurred, yet contemporary post-pandemic data on pneumococcal carriage and serotype distribution remain scarce.*

**What is New:**

• *This is the first multicenter post-COVID pneumococcal carriage study in Türkiye covering the full 0–24-year age spectrum, showing that carriage remains stable at ~20%.*

• *Direct vaccine protection against carriage is confined to children ≤10 years, with no measurable impact in adolescents or young adults. Some vaccine serotypes and non-vaccine serotypes still dominate carriage, and higher-valency PCVs would markedly improve theoretical coverage.*

## Introduction

*Streptococcus* *pneumoniae* is a gram-positive coccus responsible for a wide clinical spectrum, from mucosal disease to severe invasive infections. Globally, it remains a leading cause of community-acquired pneumonia, sepsis, and meningitis in both children and adults, with the highest incidence in children < 2 years and adults ≥ 65 years. Case-fatality rises steeply with age, particularly in low- and middle-income countries [1, 2].

Pneumococcal virulence is determined chiefly by its polysaccharide capsule. More than 100 capsular serotypes have been identified [3], and their invasive potential varies markedly [4]. Conjugate pneumococcal vaccines (PCVs), first introduced in 2000, exploit T-cell-dependent responses to reduce carriage and disease caused by vaccine serotypes [5]. After widespread PCV use, vaccine-type (VT) invasive disease, pneumonia, and otitis media have fallen sharply, especially in children < 5 years [6]. However, nasopharyngeal carriage—the reservoir for transmission—persists. Colonization begins in early infancy, peaks in preschoolers, and declines through adolescence; crowding, day-care attendance, recent antibiotic use, and chronic disease increase the odds of carriage [7–9]. The prevalence of S. pneumoniae ranged between 26.7% and 90.7% [7]. Following the PCV introduction, vaccine-type carriage falls, but non-vaccine serotypes (NVTs) often fill the ecological niche (“serotype replacement”) [8]. During the COVID-19 pandemic, non-pharmaceutical interventions suppressed respiratory pathogen spread; invasive pneumococcal disease, like many other infectious diseases, fell sharply and then rebounded with easing restrictions [10].

First licensed PCV is PCV7 (serotypes 4, 6B, 9 V, 14, 18 C, 19 F, 23 F), and then PCV10 (adds 1, 5, 7 F to PCV7) and PCV13 (adds 3, 6 A, 19 A to PCV10) have been launched [5, 11, 12]. To increase the coverage of non-vaccine-type IPD and mucosal disease, higher valency formulations have been developed and licensed in several countries: PCV15 (adds 22 F, 33 F to PCV13) and PCV20 (adds 8, 10 A, 11 A, 12 F, 15B/C to PCV15) [13]. The increase in adult cases with IPD from serotypes covered by PCVs indicates that indirect protection may be constrained. Moreover, non-vaccine serotypes and some persistent vaccine serotypes, some linked to antibiotic resistance, continue to cause invasive disease [3]. Future vaccine initiatives encompass expanding the serotype coverage in PCVs for both children and adults; even broader vaccines are in the pipeline (Table 1).

**Table 1:**
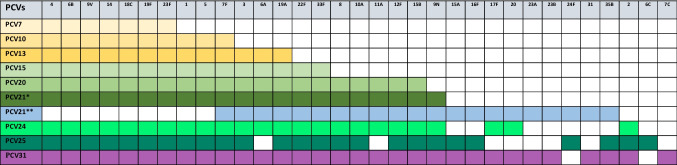
Incremental serotype composition of pneumococcal conjugate vaccines (PCVs). Rows (top → bottom) list successive licensed and pipeline vaccine formulations by *increasing valency*: PCV7, PCV10, PCV13, PCV15, PCV20, PCV21* (pediatric/broad 21-valent), PCV21** (adult-oriented alternative), PCV24, PCV25, and PCV31. Columns (left → right) list individual capsular serotypes relevant to at least one formulation: 4, 6B, 9 V, 14, 18 C, 19 F, 23 F, 1, 5, 7 F, 3, 6 A, 19 A, 22 F, 33 F, 8, 10 A, 11 A, 12 F, 15B, 9 N, 15 A, 16 F, 17 F, 20, 23 A, 23B, 24 F, 31, 35B, 2, 6 C, 7C. Filled (colored) cell—the serotype is *included* in that vaccine formulation. Blank cell—serotype is *not* included in that formulation

In Türkiye, after the introduction of PCV13 in 2011, a marked reduction in carriage rate was reported, yet both vaccine type and NVT pneumococci continue to circulate [14]. Early reports during pandemic, the nasopharyngeal *S. pneumoniae* carriage rate is higher in patients with COVID-19 than in non-infected children [15]. To date, multi-center post-pandemic data on pneumococcal carriage and serotype distribution are lacking. We therefore conducted a multicenter study to determine the prevalence of nasopharyngeal carriage and the serotype landscape among healthy children, adolescents, and young adults aged 0–24 years in the post-COVID-19 era.

## Materials and methods

### Study design and setting

We conducted a multicenter, prospective, cross-sectional study to determine the prevalence of nasopharyngeal *Streptococcus pneumoniae* carriage and its serotype distribution in healthy children, adolescents, and young adults aged 0–24 years. Recruitment was carried out between 1 April 2022 and 31 August 2022 in nine outpatient healthcare facilities located in six provinces of Türkiye (Istanbul, Eskisehir, Ankara, İzmir, Kayseri, and Bursa). The study protocol was reviewed and approved by the Clinical Research Ethics Committee of Eskisehir Osmangazi University (decision no. 64; 17 March 2022). Written informed consent was obtained from all participants or their parents/guardians. The study was conducted in accordance with the ethical standards of the institutional and national research committee and with the 1964 Helsinki Declaration and its later amendments or comparable ethical standards. The study was funded by the Scientific Research Projects Commission of Eskisehir Osmangazi University (grant TSA-2022–2445).

### Study participants

Healthy volunteers aged 0–24 years were invited to participate. For each participant, we recorded age, sex, daycare/school/university attendance, household smoking exposure, history of upper-respiratory infection within the previous month, antibiotic use within the previous 3 months, COVID-19 infection and vaccination status, and receipt of PCVs.

### Specimen collection and transport

Nasopharyngeal swabs were obtained by trained personnel. Swabs were immediately placed in 1 mL DiaRex® glycerol-buffered saline and stored at − 80 °C on site. After completion of enrolment, specimens were shipped on dry ice to the central lab for molecular analyses; temperature loggers confirmed an uninterrupted cold chain. The central laboratory was blinded to demographical findings and vaccination status.

### DNA extraction

Genomic DNA was extracted with the DiaRex® DNA Extraction Kit (BLD-5295, Diagen, Ankara, Türkiye) according to the manufacturer’s protocol. Briefly, 200 µL thawed sample was mixed with 25 µL proteinase K and 250 µL lysis buffer, incubated at 56 °C for 15 min, combined with 250 µL cold absolute ethanol, and loaded onto a spin column. After centrifugation (8000 × g, 1 min), the column was washed once with 500 µL Wash-1 and twice with 500 µL Wash-2 buffers. DNA was eluted in 70 µL elution buffer (ambient, 1 min; 8000 × g, 1 min) and stored at − 80 °C until PCR.

#### Detection of *Streptococcus pneumoniae* and serotyping

A TaqMan™ real-time PCR assay (ECD_DNZ 2010 Realtime PCR Kit; Ankara, Türkiye) was run on a Bio-Rad CFX96 system. Each 20 µL reaction contained 10 µL Mix-A, 5 µL Mix-B, and 5 µL DNA template. Cycling conditions were 95 °C × 10 s, 59 °C × 30 s (data acquisition, HEX channel), and 72 °C × 5 s for 40 cycles. The assay detects as few as 10 genome copies per reaction. Samples with threshold-cycle (CT) values ≤ 35 were deemed positive; those with > 35–40 were retested in duplicate and called positive only if reproducible [16]. Positive (100-copy plasmid) and negative (nuclease-free water) controls were included in each run. All pneumococcus-positive specimens were typed for 33 serotypes/serogroups (1, 2, 3, 4, 5, 6A/B, 6 C, 7 C, 7 F, 8, 9N/L, 9 V, 10 A, 11 A, 12 F, 14, 15 A, 15B/C, 16 F, 17 F, 18 C, 19 A, 19 F, 20, 22 F, 23 A, 23B, 23 F, 24 F, 31, 33 F, 35B, 37) using single-plex real-time PCR assays.

### Statistical analysis

Statistical analyses were performed using the JASP software (JASP Team, 2024; Version 0.19.3). Carriage prevalence was expressed as percentages. Univariate associations between pneumococcal carriage and demographic or covariate variables were tested using χ^2^ or Fisher’s exact tests, with effect sizes reported as odds ratios and 95% CIs. A multivariable mixed-effects model was explored but did not reveal significant predictors, and therefore, only univariate results are presented. A two-tailed *p* < 0.05 was considered significant.

## Results

In this study, nasopharyngeal samples were collected from 1585 children, adolescents, and young adults (797 girls, 788 boys; aged between 0 and 24 years old) from nine different centers in six major cities of Türkiye. There were 348 infants and children between the ages of 0–5 years old (22.0%), 464 children (29.3%) between the ages of 6–10 years, 267 children (16.8%) between the ages of 11–15 years, 205 adolescents (12.9%) between the ages of 16–18 years, and 301 young adults (19.0%) between the ages of 19–24 years. Among the 0–5 years-old group, 20.7% attended day care centers. In the 6–18 years of age group, 85.6% were in school; in the 19–24 groups, 88.6% were attending university. Nine of the participants, who were of age eligible for military service, had done their compulsory military service. In total, 784 cases (49.5%) had indoor smoking exposure, and 245 (15.4%) had active smoking; 795 (50.1%) cases had a history of upper respiratory tract infection (URTI) within the past month; 376 (23.7%) cases had used antibiotics within the last month, and 680 (42.9%) cases had used antibiotics within the past 3 months; 103 (6.4%) participants had a history of PCR-positive COVID-19 infection within the past year, and 267 (16.8%) cases had a history of a household member with COVID-19 within the past year; 420 participants (26.5%) have a previous history of at least one dose of the COVID-19 vaccine. Two-thirds of participants had received a PCV (1049/1585, 66.2%). Uptake exceeded 80% in all children ≤ 15 years, fell to 44% in 16–18 years and 13% in 19–24 years.

**Fig. 1:**
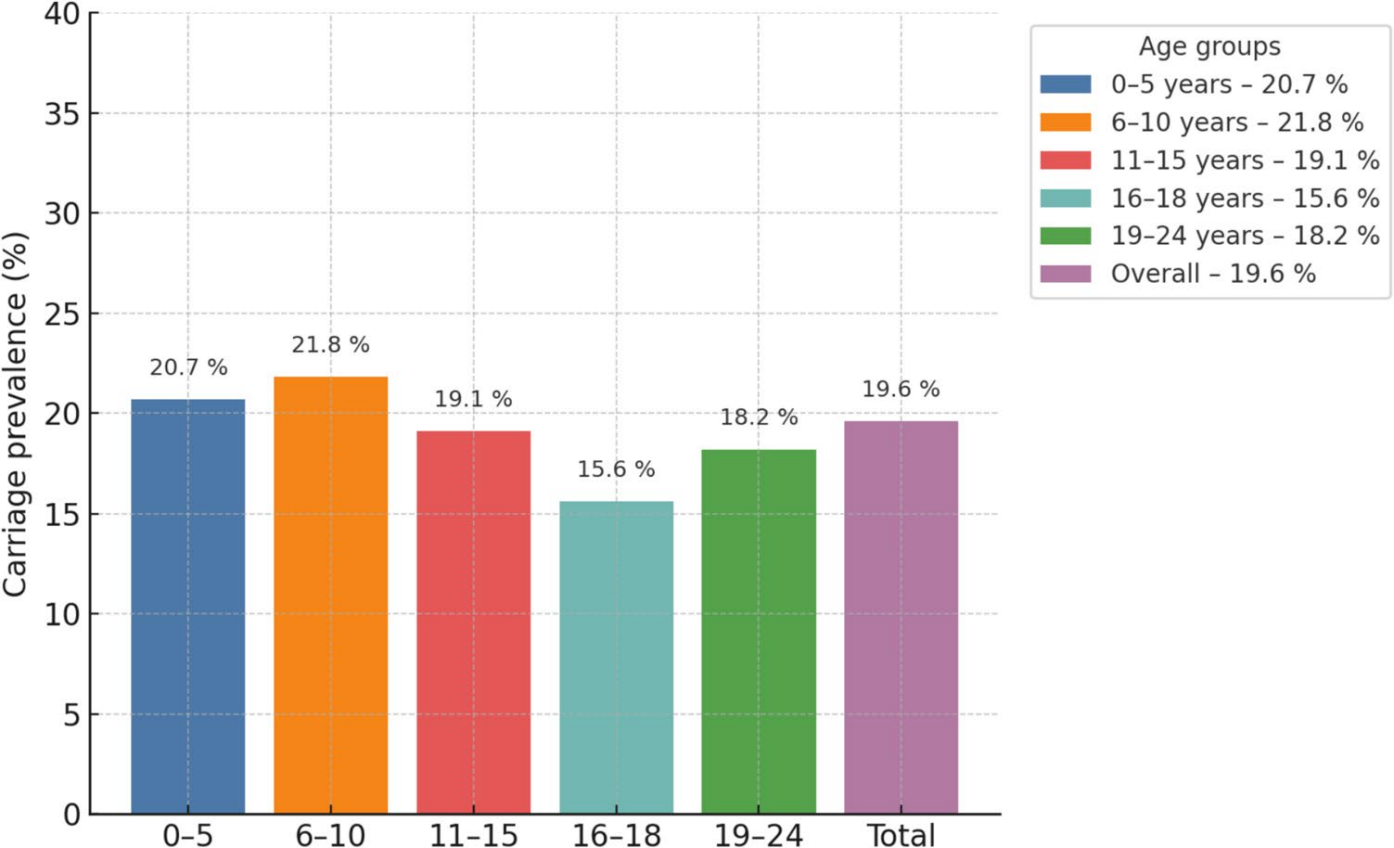
Age‑specific prevalence of nasopharyngeal *Streptococcus pneumoniae* carriage in Türkiye, 2022. Vertical bars show the proportion of carriers in each 5‑year age stratum (0–5, 6–10, 11–15, 16–18, 19–24 years) and in the overall cohort (Total, *n* = 1585). Exact prevalence is printed above each bar

Among the 1585 healthy participants aged 0–24 years, 311 harbored *Streptococcus pneumoniae*, giving an overall nasopharyngeal pneumococcal carriage prevalence of 19.6%. Carriage rate according to the age groups has been shown in Fig. 1. Carriage was highest in the 6–10-year age group at 21.8% (101/464) and lowest in adolescents aged 16–18 years at 15.6% (32/205). The prevalence in the 0–5-year (20.7%; 72/348), 11–15-year (19.1%; 51/267), and 19–24-year (18.2%; 55/301) groups was intermediate and quite similar to the cohort average.

**Fig. 2:**
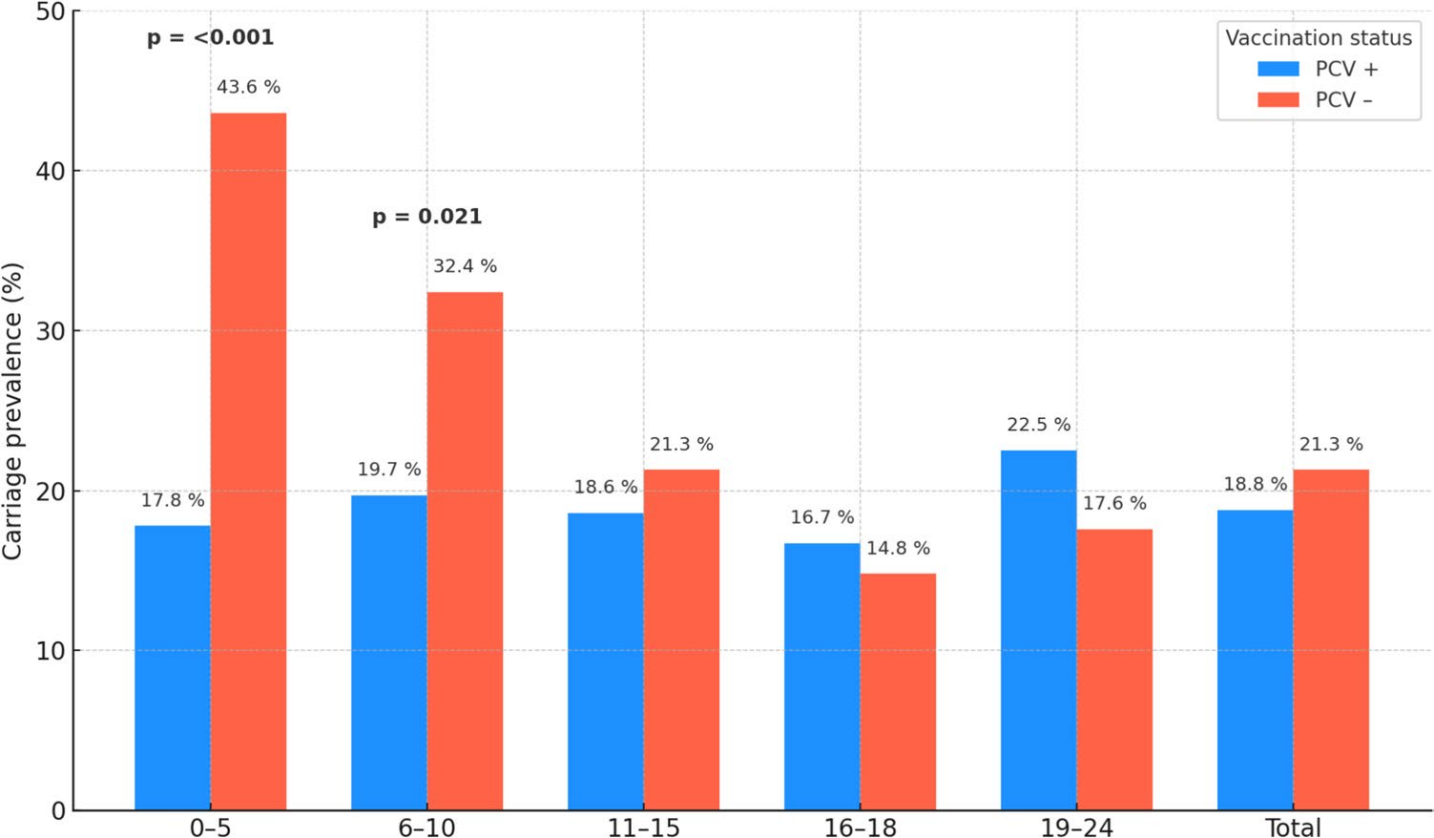
Carriage prevalence by pneumococcal‑conjugate‑vaccine (PCV) status and age group. Grouped bars compare PCV‑vaccinated (blue) and unvaccinated (orange) participants within each age stratum and overall. Percentages are annotated above each bar. Statistically significant differences (*χ*.^2^ test) are flagged with bold *p*‑values above the corresponding bar clusters (0–5 years, *p* < 0.001; 6–10 years, *p* = 0.021)

Two-thirds of the entire cohort (66.2%) have received at least one dose of PCV. PCV coverage approaches 90% in the 0–5-year group (88.8%) and exceeds 80% in both 6–10 years (84.1%) and 11–15-year (82.4%) cohorts. Coverage drops sharply after mid-adolescence; only 43.9% of 16–18-year-olds and 13.3% of 19–24-year-olds are vaccinated. Pneumococcal carriage prevalence by PCV status and age group is summarized in Table 2 and Fig. 2. Significant protection was restricted to children ≤ 10 years, with 49–72% lower odds of carriage among vaccinees. No effect was detected in older age groups. In the 0–5-year-old group, the pneumococcal carriage rate was 17.8% in vaccinated with PCVs group, while 43.6% in the non-vaccinated group (OR 0.28; 95% CI 0.13–0.60; *p* < 0.001). In the 6–10-year-old group, the pneumococcal carriage rate was 19.7% in vaccinated with PCVs group, while 32.4% in the non-vaccinated group (OR 0.51; 95% CI 0.28–0.93; *p* < 0.05). In the pediatric age group (0–18 years age group), the pneumococcal carriage rate was 18.6% in vaccinated with PCVs group, while 24.7% in the non-vaccinated group (OR 0.69; 95% CI 0.50–0.97; *p* < 0.05).

**Table 2:** Nasopharyngeal carriage prevalence by pneumococcal‑conjugate‑vaccine (PCV) status and age group

Age group (y)	Vaccinated with PCV within age group %	Carriage PCV +	Carriage PCV −	Odds ratio (95% CI)	*p*
0–5	**88.8%**	55/309 (17.8%)	17/39 (43.6%)	0.28 (0.13–0.60)	** < 0.001**
6–10	**84.1%**	77/390 (19.7%)	24/74 (32.4%)	0.51 (0.28–0.93)	**0.021**
11–15	**82.4%**	41/220 (18.6%)	10/47 (21.3%)	0.84 (0.37–2.07)	0.685
16–18	**43.9%**	15/90 (16.7%)	17/115 (14.8%)	1.15 (0.50–2.63)	0.847
19–24	**13.3%**	9/40 (22.5%)	46/261 (17.6%)	1.35 (0.53–3.17)	0.666
All ages	**66.2%**	197/1049 (18.8%)	114/536 (21.3%)	0.85 (0.65–1.12)	0.256

^*^PCV, pneumococcal conjugated vaccine

A univariate comparison of 311 carriers with 1 274 non‑carriers found no statistically significant associations for the demographic or exposure variables captured in the case‑report form. Carriage prevalence was virtually identical in girls (154 carriers among 797; 19.3%) and boys (160/788; 20.3%; *χ*^2^ = 0.29, *p* = 0.59). Children reporting an URTI in the preceding month (170/795; 21.4%) were colonized at the same rate as those without URTI (143/667; 21.4%; *p* = 0.99), regarding antibiotic exposure, within 1 month, 78/376 (20.7%) vs 235/1086 (21.6%) among those without recent antibiotics (*p* = 0.70), and within 3 months, 148/680 (21.8%) vs 164/782 (21.0%) (*p* = 0.67). Regarding previous COVID-19 vaccine use, carriage in vaccinated participants (85/420; 20.2%) did not differ from the unvaccinated (228/1 042; 21.9%; *p* = 0.43). Previous COVID-19 infection in the past year and household COVID-19 infection in the past year were also similar between the carriers and non-carriers. Regarding household smoking, nasopharyngeal carriage was 20.2% (158/784) when ≥ 1 household member smoked versus 22.8% (154/677) in smoke‑free homes (*p* = 0.21). Among preschoolers, carriage in day‑care attendees (16/77; 20.8%) was almost identical to those cared for at home (56/271; 20.7%). In school‑age strata (6–15 years), primary‑school pupils showed a marginally higher prevalence (90/363; 24.8%) than their same‑age peers outside formal schooling (16–22%), but differences were non‑significant (all *p* > 0.2). Carriage in university students (7/34; 20.6%) mirrored the rate in non‑student young adults (50/263; 19.0%). Despite adequate power to detect the large vaccine effect seen in ≤ 10‑year‑olds, our dataset shows no measurable influence of sex, recent viral illness, antibiotic exposure, COVID‑19-related factors, household smoking, or educational setting on pneumococcal carriage prevalence. The uniformly flat associations suggest that—in the current post‑pandemic, high‑PCV‑uptake environment—carriage risk is driven more by age‑related biological factors and serotype ecology than by short‑term behavioral or environmental exposures.

**Fig. 3:**
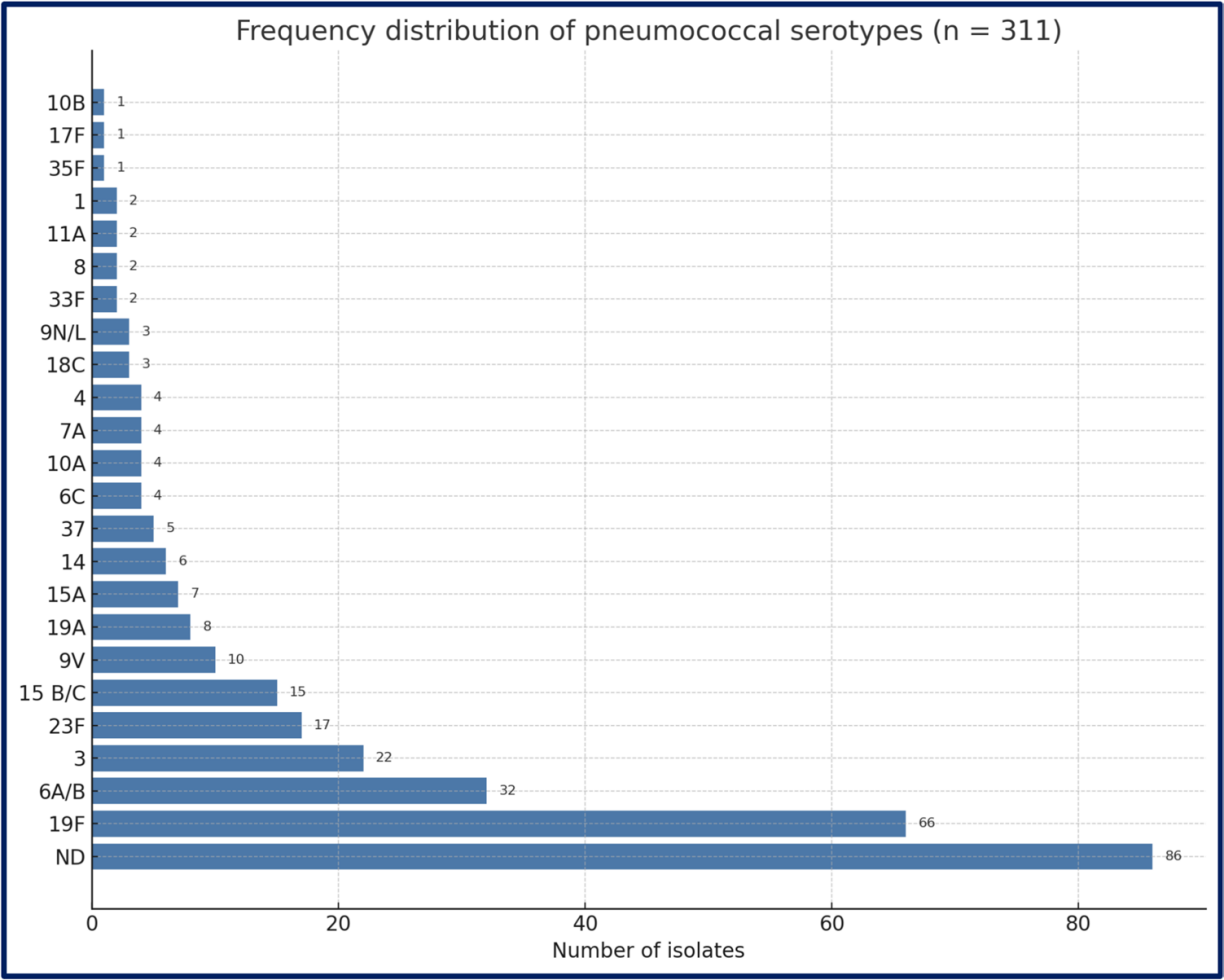
Frequency distribution of pneumococcal serotypes isolated from 311 carriers. Horizontal bars rank all serotypes detected (≥ 1 isolate) plus the ND in descending order of frequency. Bar length corresponds to the absolute isolate count; counts are printed at bar termini. Data combine all age groups. ND, not-defined

**Fig. 4:**
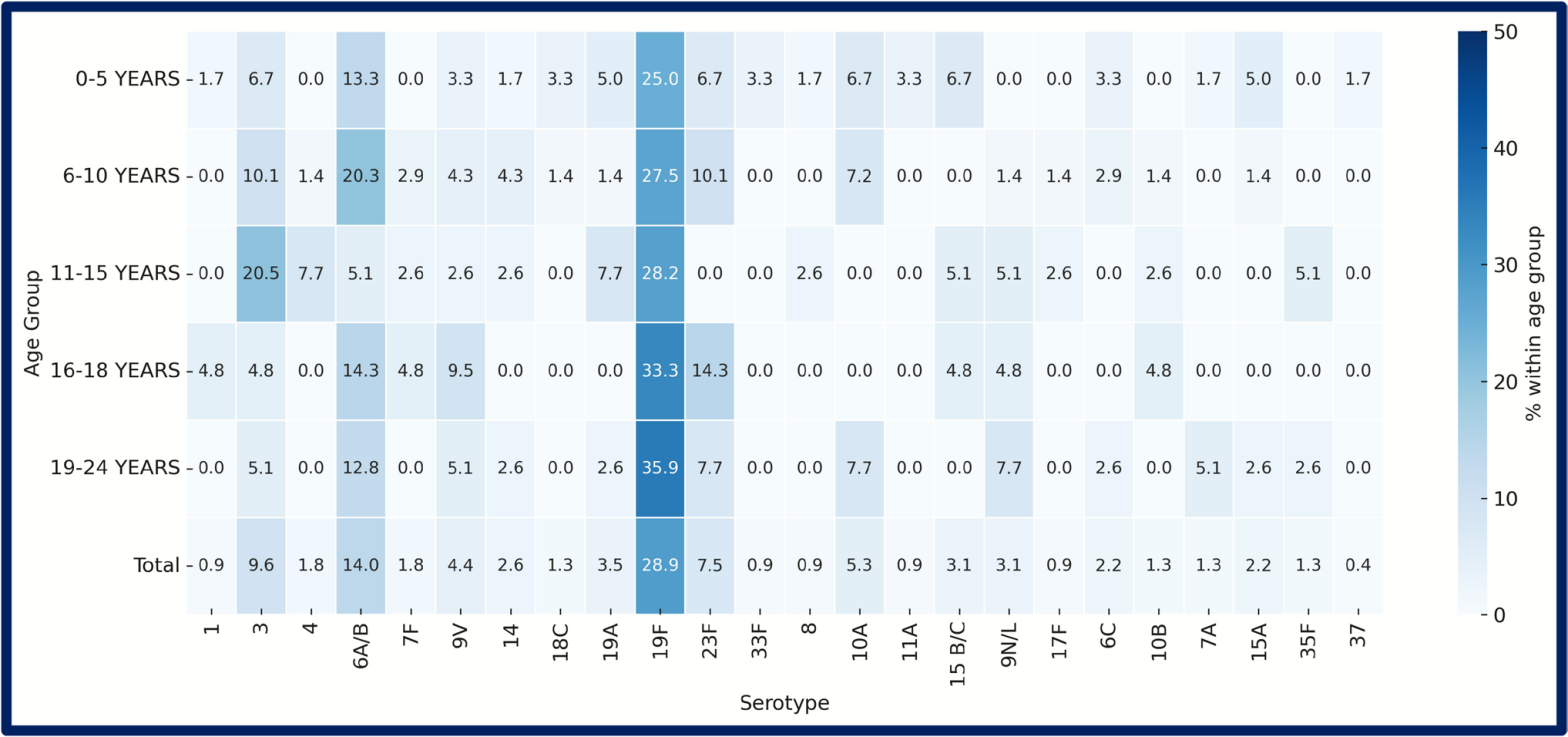
Heat‑map legend of serotype distribution according to age groups. Rows: 0–5 years, 6–10 years, 11–15 years, 16–18 years, 19–24 years: age strata of study participants. Columns (left to right): numeric serotypes listed in ascending order represent the percentage of that age group’s isolates belonging to the given serotype; deeper blue indicates higher prevalence. In‑cell numbers show the exact percentage to one decimal place; blank cells (white) denote 0%

Of 311 pneumococcal isolates recovered, 225 (72.4%) were successfully typed into 27 serotypes; 86 (27.6%) were not defined (ND). Among serotyped isolates (*n* = 225), 19 F was by far the most common, accounting for 66 isolates (29.3%). 6A/B (*n* = 32; 14.2%) and serotype 3 (*n* = 22; 9.7%) ranked second and third. Other frequent types were 23 F (*n* = 17; 7.6%), 15B/C (*n* = 15; 6.7%), 9 V (*n* = 8; 3.5%), and 19 A (*n* = 8; 3.5%). A further 18 serotypes were each found in ≤ 8 isolates, illustrating a long tail of low-frequency types (Figs. 3, 4). Twelve PCV13‐included serotypes (1, 3, 4, 5, 6A/B, 7 F, 9 V, 14, 18 C, 19 A, 19 F, 23 F) represented 174/225 isolates (77.3).

PCV7 and PCV10 would protect 53–55% in children below 5 years of age, 61–64% in all study groups of all serotyped isolates. Adding serotypes 3, 6 A, and 19 A in PCV13 lifts overall coverage to 77.3% in all study groups and 66.7% in children below 5 years of age. The frequency of PCV13 serotypes in children vaccinated with PCV13 was significantly lower than in unvaccinated children. PCV15 (adds 22 F, 33 F) yields only a fractional bump to 78.2% overall and 70% in children below 5 years of age group. PCV20 increases coverage to 88.4% overall, surpassing 84% in every age group. PCV21 (pediatric formulation) adds minimal benefit beyond PCV20 (89.8% overall). PCV24 improves coverage to 90.2%, while PCV25 and PCV31 top the list at 93.3–93.8% overall and 93.3% in children below 5 years. Because it omits 19 F, 6B, and 23 F, the adult formulation (our population only 19–24 age group) of PCV21 covers only 45.3%. The youngest cohort (0–5 years) gains the most from higher valencies, climbing from 53.3% (PCV7) to 93.3% (PCV25/31). Coverage in adolescents (11–15 years) lags behind children but still rises steadily with valency (Table 3).

**Table 3:** Theoretical coverage of current and pipeline PCVs among serotyped isolates (*n* = 225)

Vaccine	0–5 y (*n* = 60)	6–10 y (*n* = 69)	11–15 y (*n* = 39)	16–18 y (*n* = 21)	19–24 y (*n* = 36)	Overall (*n* = 225)
PCV 7	32/60 53.3%	48/69 69.6%	18/39 46.2%	15/21 71.4%	25/36 69.4%	**138/225** 61.3%
PCV 10	33/60 55.0%	50/69 72.5%	19/39 48.7%	17/21 80.9%	25/36 69.4%	**144/225** 64.0%
PCV 13	40/60 66.7%	58/69 84.1%	30/39 76.9%	18/21 85.7%	28/36 77.8%	**174/225** 77.3%
PCV 15	42/60 70.0%	58/69 84.1%	30/39 76.9%	18/21 85.7%	28/36 77.8%	**176/225** 78.2%
PCV 20	53/60 88.3%	63/69 91.3%	33/39 84.6%	19/21 90.4%	31/36 86.1%	**199/225** 88.4%
PCV 21*	53/60 88.3%	63/69 91.3%	35/39 89.7%	20/21 95.2%	31/36 86.1%	**202/225** 89.8%
PCV 21**	31/60 51.7%	30/69 43.5%	21/39 53.8%	7/21 33.3%	13/36 36.1%	**102/225** 45.3%
PCV 24	53/60 88.3%	63/69 91.3%	36/39 92.3%	20/21 95.2%	31/36 86.1%	**203/225** 90.2%
PCV 25	56/60 93.3%	65/69 94.2%	36/39 92.3%	20/21 95.2%	34/36 94.4%	**211/225** 93.8%
PCV 31	56/60 93.3%	64/69 92.8%	37/39 94.8%	20/21 95.2%	33/36 91.7%	**210/225** 93.3%

Serotype coverage of all vaccines is shown in Table 1. PCV21 vaccine candidates do not include the same serotypes: PCV21* (pediatric/broad 21-valent), PCV21** (adult-oriented alternative)

## Discussion

This multicenter survey provides the first post‑COVID-19 snapshot of nasopharyngeal pneumococcal carriage across the full 0–24‑year age span in Türkiye. Using a harmonized protocol (standardized swabbing, central laboratory PCR detection, and single‑plex serotyping), overall carriage prevalence was 19.6%, peaking at 20.85 and 21.8% in children aged 0–5 years and 6–10 years, respectively. These results lie within the 17–25% range reported for Turkish children in the PCV13 era and parallel the 19–33% observed in the UK [15, 17, 18].

Among the 225 serotyped pneumococcal isolates, serotype 19 F accounted for 29.3%, followed by 6A/B, serotype 3, 23 F, and 15B/C. Syrogiannopoulos and colleagues [16] determined that pneumococcal carriage was 48.6% in 1212 children in Greece after routine PCV13 vaccination using molecular methods. The most common serotypes detected in healthy children with pneumococcal carriage were serotype 3, serotype 19 F, serotype 9A/L/N/V, serotype 15A/B/C/F, serotype 11 A, serotype 19 A, and serotype 7A/F [16]. In Türkiye, Kanık-Yüksel et al. [14] also found serotypes 3, 19 F, 6A/6B, 11 A, and 15B to be the most common in a carriage study of healthy children. Ceyhan and colleagues [18] found that out of 580 healthy children under 5 years old tested between 2019 and 2020, 17.8% carried pneumococcus, with the most common types being serotype 15B, serotype 23 F, serotype 23 A, serotype 11 A, serotype 19 F, and serotype 15F. There is increasing concern around vaccine types still capable of causing severe disease despite being included in PCVs, particularly serotypes 3, 19 A, and 19 F [3]. Serotypes such as 19 F and 19 A have persisted despite their inclusion in PCVs. The persistence of 19 F after 13 years of PCV13 use mirrors reports from other studies of capsule switching or clonal expansion of partially vaccine-escape lineages. Serotype 3 persistence could be due to the bacterium’s thick capsule surrounded by a mucoid layer or due to capsule polysaccharide ejection from the synthase [3]. Persistent or little decline serotype 3 IPD and carriage in the USA, clade II has increased since 2010 and this might be related to breakthrough infection [19].

There have been changes in the epidemiology of other infectious diseases and routine/risk group vaccination rates worldwide following COVID-19 infection [10]. Horvath et al. [20] evaluated pneumococcal carriage in 401 children attending day care centers in Hungary who were screened between April 2022 and April 2023. Overall, the carriage rate was 16.5%, mainly non-PCV13 serotypes, and serotypes 23B, 35 F, 15A/F, 15B/C, 11 A, and 23 A were most prevalent. In 2020, a study conducted by Aykaç et al. [15] in our country found that 32.2% of children who tested positive for COVID-19 were carriers of *S. pneumoniae*. Our study was conducted in the third year of the pandemic, during a period when pandemic restrictions were eased, and during this period, there were changes in the epidemiology of other viral infections in children alongside COVID-19 infection.

Two-thirds of the entire cohort (66.2%) have received at least one dose of PCV. In Türkiye, the PCV7 was first added to the National Immunization Schedule in 2008, with a 3 + 1 dose schedule at 2, 4, 6, and 12 months of age. In 2011, the PCV13 was included in the national immunization schedule under the same schedule. In 2020, PCV13 was switched to a 2 + 1 dose schedule (2nd, 4th, and 12th months). In addition to this, PCVs are also administered to risk groups and individuals over the age of 65 in our country. The vaccination rate with PCVs (PCV13) was 88.8% in the 0–5 age group, and in this age group, some infants were vaccinated according to the 2 + 1 schedule between 2020 and 2022. In this group, there were infants younger than 2 months who had not yet received the first dose of the pneumococcal vaccine. Pneumococcal carriage was found to be significantly lower in unvaccinated individuals in the 0–5 age group (72% reduction) and the 6–9 age group (49% reduction). The frequency of PCV13 serotypes in children vaccinated with PCV13 was significantly lower than in unvaccinated children. In the 11 years and older age group, there were children and adolescents who were unvaccinated because PCV7, PCV13, and the vaccine had not yet been included in the vaccination schedule.

Following the widespread use of PCVs, there has been a decrease in the incidence of acute otitis media, otitis-related complications, antibiotic use, pneumonia, and meningitis (especially in vaccine serotypes) in children. Although there has been a decrease in the burden of disease following vaccination, carriage of serotypes not covered by current vaccines and diseases associated with these serotypes have been reported [6]. Although higher-valency PCVs increase theoretical strain coverage, there is a potential trade-off between breadth and depth of immune response. Increasing antigen content can modestly reduce immunogenicity for certain serotypes. Pre-licensure data suggest that while PCV15 and PCV20 generally achieve non-inferiority versus PCV13 for shared serotypes, opsonophagocytic activity for some antigens may be numerically lower [21]. Previous reports also showed better immunogenicity for serotype 3 with PCV15 [22]. The effect of PCV15 for serotype 3 carriage would be critical to estimate the real-world vaccine effectiveness of PCV15. Whether these differences will affect clinical protection, particularly for immunologically challenging serotypes such as serotype 3, remains to be clarified through post-licensure effectiveness studies and ongoing surveillance. Further studies are needed to evaluate protection against additional serotypes for other PCVs in the pipeline.

The standard method for evaluating pneumococcal carriage is culture and serotype determination using antisera. However, the use of traditional culture-based methods may result in the overlooking of serotypes with lower prevalence. Therefore, with the recent development of molecular diagnostic methods, significant progress has been made in the detection and serotyping of pneumococcal pathogens (16). Direct molecular methods are particularly important in situations where antibiotic use is widespread in respiratory tract infections in the community. In our study, molecular methods were used for pneumococcal identification and serotyping in nasopharyngeal samples. This is the largest Turkish dataset since PCV introduction, spanning nine centers and > 1500 participants. Broad age continuum captures school‑age and young‑adult reservoirs rarely studied. Post‑pandemic timing fills a global gap on how carriage rebounded after non‑pharmaceutical interventions were relaxed. We have some limitations. Cross‑sectional design precludes assessment of acquisition dynamics, persistence, or seasonality. Collapsed serotype groups (6A/B, 15B/C) mask differential vaccine match for 6 A, 6B, 15 A, and 15C.

In Türkiye, nasopharyngeal pneumococcal carriage remained common (~ 20%) across childhood. PCV immunization was associated with a significant reduction in carriage in 0–18 years old, especially among children ≤ 10 years, reflecting both high vaccine uptake and dominance of vaccine serotypes in that age band. Although there has been a decrease in the pneumococcal carriage following vaccination, carriage of some vaccine and non-vaccine serotypes has been observed. Higher‑valency PCVs and enhanced genomic serotype surveillance are needed to address residual carriage and guide future immunization strategies.

## Data Availability

No datasets were generated or analysed during the current study.

## References

[1] Feldman C, Anderson R (2020) Recent advances in the epidemiology and prevention of *Streptococcus pneumoniae* infections. F1000Res 9:F1000 Faculty Rev-338. 10.12688/f1000research.22341.1 32411353 10.12688/f1000research.22341.1 PMC7212261

[2] Yildirim I, Shea KM, Pelton SI (2015) Pneumococcal disease in the era of pneumococcal conjugate vaccine. Infect Dis Clin North Am 29(4):679–697. 10.1016/j.idc.2015.07.009 26610421 10.1016/j.idc.2015.07.009 PMC4662776

[3] Ramos B, Vadlamudi NK, Han C, Sadarangani M (2025) Future immunisation strategies to prevent *Streptococcus pneumoniae* infections in children and adults. Lancet Infect Dis 25(6):e330–e344. 10.1016/S1473-3099(24)00740-0 40112854 10.1016/S1473-3099(24)00740-0

[4] Metcalf BJ, Waldetoft KW, Beall BW, Brown SP (2023) Variation in pneumococcal invasiveness metrics is driven by serotype carriage duration and initial risk of disease. Epidemics 45:100731. 10.1016/j.epidem.2023.100731 38039595 10.1016/j.epidem.2023.100731 PMC10786323

[5] Dinleyici EC, Yargic ZA (2008) Pneumococcal conjugated vaccines: impact of PCV-7 and new achievements in the postvaccine era. Expert Rev Vaccines 7(9):1367–1394. 10.1586/14760584.7.9.1367 18980540 10.1586/14760584.7.9.1367

[6] Chapman R, Sutton K, Dillon-Murphy D, Patel S, Hilton B, Farkouh R, Wasserman M (2020) Ten year public health impact of 13-valent pneumococcal conjugate vaccination in infants: a modelling analysis. Vaccine 21(45):7138–7145. 10.1016/j.vaccine.2020.08.068 10.1016/j.vaccine.2020.08.068 32912642

[7] Tvedskov ESF, Hovmand N, Benfield T, Tinggaard M (2022) Pneumococcal carriage among children in low and lower-middle-income countries: a systematic review. Int J Infect Dis 115:1–7. 10.1016/j.ijid.2021.11.021 34800691 10.1016/j.ijid.2021.11.021

[8] Klugman KP, Rodgers GL (2023) Pneumococcal carriage and seroepidemiology studies to measure current and future pneumococcal conjugate vaccine effectiveness. J Infect Dis 227(1):608–609. 10.1093/infdis/jiac377 36130329 10.1093/infdis/jiac377

[9] Simell B, Auranen K, Käyhty H, Goldblatt D, Dagan R, O'Brien KL; Pneumococcal carriage group (2012) The fundamental link between pneumococcal carriage and disease. Expert Rev Vaccines 11(7):841–55. 10.1586/erv.12.53 10.1586/erv.12.53 22913260

[10] Dinleyici EC, Borrow R, Safadi MAP, van Damme P, Munoz FM (2021) Vaccines and routine immunization strategies during the COVID-19 pandemic. Hum Vaccin Immunother 17(2):400–407. 10.1080/21645515.2020.1804776 32845739 10.1080/21645515.2020.1804776 PMC7899627

[11] Dinleyici EC, Yargic ZA (2009) Pneumococcal conjugated vaccine: PHiD-CV. Expert Rev Anti Infect Ther 7(9):1063–1074. 10.1586/eri.09.84 19883326 10.1586/eri.09.84

[12] Dinleyici EC, Yargic ZA (2009) Current knowledge regarding the investigational 13-valent pneumococcal conjugate vaccine. Expert Rev Vaccines 8(8):977–986. 10.1586/erv.09.68 19627181 10.1586/erv.09.68

[13] Kobayashi M, Farrar JL, Gierke R, Britton A, Childs L, Leidner AJ, Campos-Outcalt D, Morgan RL, Long SS, Talbot HK, Poehling KA, Pilishvili T (2022) Use of 15-valent pneumococcal conjugate vaccine and 20-valent pneumococcal conjugate vaccine among U.S. adults: updated recommendations of the advisory committee on immunization practices - United States, 2022. MMWR Morb Mortal Wkly Rep 71(28):109–117. 10.15585/mmwr.mm7104a1 35085226 10.15585/mmwr.mm7104a1 PMC9351524

[14] Kanık Yüksek S, Tezer H, Gülhan B, Özkaya Parlakay A, Güldemir D, Coskun-Ari FF, Bedir Demirdağ T, Kara Uzun A, Kızılgün M, Solmaz S, Kılıç S, Yalınay Çırak M, Baran Aksakal FN (2020) Nasopharyngeal pneumococcal carriage in healthy Turkish children after 13-valent conjugated pneumococcal vaccine implementation in the national immunization program. J Infect Public Health 13(2):266–274. 10.1016/j.jiph.2019.10.009 31818710 10.1016/j.jiph.2019.10.009

[15] Aykac K, Ozsurekci Y, Cura Yayla BC, Evren K, Lacinel Gurlevik S, Oygar PD, Yucel M, Karakoc AE, Alp A, Cengiz AB, Ceyhan M (2021) Pneumococcal carriage in children with COVID-19. Hum Vaccin Immunother 17(6):1628–1634. 10.1080/21645515.2020.1849516 33449815 10.1080/21645515.2020.1849516 PMC8115562

[16] Syrogiannopoulos GA, Grivea IN, Moriondo M, Nieddu F, Michoula AN, Calabrese MR, Anthracopoulos M, Azzari C (2021) Molecular surveillance of pneumococcal carriage following completion of immunization with the 13-valent pneumococcal conjugate vaccine administered in a 3 + 1 schedule. Sci Rep 11(1):24534. 10.1038/s41598-021-03720-y 34969968 10.1038/s41598-021-03720-y PMC8718523

[17] Cleary DW, Campling J, Lahuerta M, Hayford K, Southern J, Gessner BD, Lo SW, Bentley SD, Faust SN, Clarke SC (2024) Non-pharmaceutical interventions for COVID-19 transiently reduced pneumococcal and Haemophilus influenzae carriage in a cross-sectional pediatric cohort in Southampton, UK. Microbiol Spectr 12(8):e0022424. 10.1128/spectrum.00224-24 38990033 10.1128/spectrum.00224-24 PMC11302307

[18] Ceyhan M, Karadag-Oncel E, Hascelik G, Ustundag G, Gurbuz V, Samlioglu P, Yilmaz N, Ozsurekci Y, Yilmaz E, Aykac K, Oz FN, Uzum O, Orsdemir-Hortu H, Tanir G, Yilmaz-Ciftdogan D, Kurugol Z (2021) Nasopharyngeal carriage of *Streptococcus pneumoniae* in healthy children aged less than five years. Vaccine 39(8):2041–2047. 10.1016/j.vaccine.2021.03.028 33741188 10.1016/j.vaccine.2021.03.028

[19] Cella E, Sutcliffe CG, Grant LR, Tso C, Weatherholtz RC, Littlepage S, Becenti L, Jubair M, Simons BC, Harker-Jones M, Reid R, Yazzie D, Santosham M, O’Brien KL, Hammitt LL, Azarian T (2024) *Streptococcus pneumoniae* serotype 3 population structure in the era of conjugate vaccines, 2001–2018. Microb Genom 10(3):001196. 10.1099/mgen.0.001196 38498591 10.1099/mgen.0.001196 PMC10963907

[20] Horváth A, Huber A, Bartha Á, Hajósi-Kalcakosz S, Kristóf K, Dobay O (2025) Pneumococcal carriage among young children attending daycare in Hungary, 12–13 years post-PCV13: a cross-sectional study. Sci Rep 15(1):22696. 10.1038/s41598-025-07777-x 40593220 10.1038/s41598-025-07777-x PMC12215634

[21] De Wals P (2024) PCV13, PCV15 or PCV20: Which vaccine is best for children in terms of immunogenicity? Can Commun Dis Rep 50(1–2):35–39. 10.14745/ccdr.v50i12a04 38655244 10.14745/ccdr.v50i12a04 PMC11037880

[22] Wagner G, Gartlehner G, Thaler K, Ledinger D, Feyertag J, Klerings I, Saif-Ur-Rahman KM, Devane D, Olsson K, Adel Ali K, Vygen-Bonnet S, Salo H, Zavadska D, Grgič Vitek M, Oona M, Cunney R, Tuerlinckx D, Kristensen Lomholt F, Sommer I (2024) Immunogenicity and safety of the 15-valent pneumococcal conjugate vaccine, a systematic review and meta-analysis. NPJ Vaccines 9(1):257. 10.1038/s41541-024-01048-y 39738219 10.1038/s41541-024-01048-y PMC11685527

